# Supplementary material for: The Dose-Related Efficacy of Human Placenta-Derived Mesenchymal Stem Cell Transplantation on Antioxidant Effects in a Rat Model with Ovariectomy
Source: Antioxidants (Basel). 2023 Aug 7;12(8):1575. doi: 10.3390/antiox12081575 (PMC10451747; doi:10.3390/antiox12081575)
Supplement: Supplementary file 1 [file antioxidants-12-01575-s001.zip › antioxidants-2515444-supplementary.pdf]

Supplementary Materials

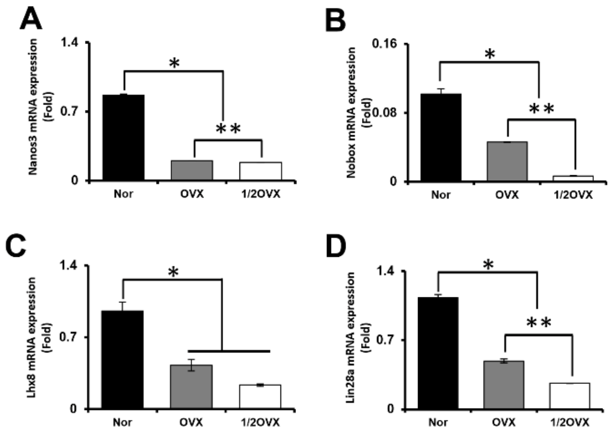

Figure S1. Comparison of OVX rat ovarian tissues and 1/2 OVX rat ovarian tissues. The mRNA expression of Nanos3 (**A**), Nobox (**B**), Lhx8 (**C**), and Lin28a (**D**) in ovarian tissues was analyzed by qRT–PCR. Significance at $p<0.05$ is indicated by an asterisk (*) for the normal group vs. the other groups and (**) for the OVX group vs. the 1/2 OVX group.

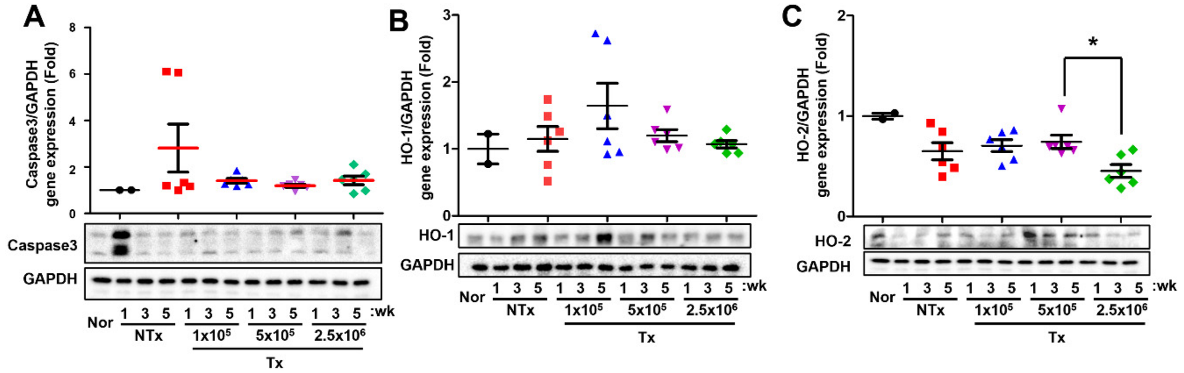

Figure S2. Effect of PD-MSC cocultivation on oxidative stress. The gene expression of Caspase 3 (**A**), HO-1 (**B**), and HO-2(**C**) in the ovarian tissues of 1/2 OVX rats 1, 3, and 5 weeks after transplantation was analyzed by western blotting. Significance at $p<0.05$ is indicated by an asterisk (*).

Table S1. Primer sequences using quantitative real-time polymerase chain reactions were used in this study.

Gene	Sequence	Accession number
**Human Alu**	F: 5'- GGA GGC TGA GGC AGG AGA A-3'<br>R: 5'- CGG AGT CTC GCT CTG TCG CCC A-3'	002715
**Rat Nanos3**	F: 5'- CTCTGCATGAGGAAGAGGAGCC-3'<br>R: 5'- GGACTGATAGATCGCACGAGA-3'	031541.2
**Rat Lhx8**	F: 5'- GTATCACTTGGCTTGCTT-3'<br>R: 5'- ATTACCGTTCTCCACTTC-3'	017286.3
**Rat Lin28a**	F: 5'- CCCGGTGGACGTCTTTGTG-3'<br>R: 5'- CACTGCCTCACCCTCCTTGA-3'	012753.3
**Rat Nobox**	F: 5'- AGCCAGTGCAGATCTGCACCG-3'<br>R: 5'- TGTCACTGCCAGGAACATCCCTC-3'	001042619.1
**Rat BMP15**	F: 5'- ATC TGA TGT CCC TTG TCC TT-3'<br>R: 5'- CTC TGT ATG ATG GCA TGG TT-3'	017008.4
**Rat EGFR**	F: 5'-AGA TTG CAA AGG GCA TGA ACT AC-3'<br>R: 5'-ACA TTC CTG GCT GCC AAG TG-3'	039091618.1
**Rat NOX4**	F: 5'-AGG TGT CTG CAT GGT GGT G-3'<br>R: 5'- GAG GGT GAG TGT CTA AAT TGG T -3'	053524.1
**Rat P4hb**	F: 5'-AGC TGC CTT TGG TCA TCG AG-3'<br>R: 5'-AGT ATG CGC TGG TTG TCA GT-3'	012998.2
**Rat HO1**	F: 5'-GCG AAA CAA GCA GAA CCC A -3'<br>R: 5'-GCT CAG GAT GAG TAC CTC CCA -3'	012580.2
**Rat HO2**	F: 5'-AGG GCA GCA CAA ACA ACT CA-3'<br>R: 5'-TCT GGC TCA TTC TGT CCT AC-3'	039086914.1
**Rat SOD1**	F: 5'-TTT TGC TCT CCC AGG TTC CG-3'<br>R: 5'-TGT CCT GAC ACC ACA ACT GG-3'	017050.1
**Rat SOD2**	F: 5'-AGC TGC ACC ACA GCA AGC AC-3'<br>R: 5'-TCC ACC ACC CTT AGG GCT CA-3'	017051.2
**Rat SIRT7**	F: 5'-CAG GTG TCA CGC ATC CTG AG-3'<br>R: 5'-GCC CGT GTA GAC AAC CAA GT-3'	001107073.1
**Rat PGC1α**	F: 5'-TTG CCC AGA TCT TCC TGA AC-3'<br>R: 5'-TGA GGA CCG CTA GCA AGT TT-3'	031347.1
**Rat GAPDH**	F: 5'-TCC CTC AAG ATT GTC AGC AA-3'<br>R: 5'-AGA TCC ACA ACG GAT ACA TT-3'	017008.4

Table. S2 The study of stem cell therapy on ovarian failure mouse and rat model

Organism	Stem Cell type	Transplantation Route	Stem Cells Quantity for transplantation	References
Mouse	ADSC	I.V.	1x10^4	[1]
Mouse	ADSC	I.V.	1x10^6	[2]
Mouse	AEC	I.P.	4x10^6	[3], NCT02912104
Mouse	AEC	I.V.	2x10^6	[4, 5] NCT03207412
Mouse	AEC	Local	1x10^3	[6]
Mouse	AEC	Local	2x10^4	[7]
Mouse	AFSC	Local	1x10^3	[8]
Mouse	AFSC	Local	2-5x10^3	[9]
Mouse	AFSC	Local	5x10^5	[10]
Mouse	AMSC	I.P.	2x10^6	[11]
Mouse	AMSC	Local	1x10^6	[12]
Mouse	BMMSC	I.P.	5x10^5	[13]
Mouse	BMMSC	I.V.	5x10^5	[14, 15]
Mouse	BMMSC	I.V.	1x10^6	[16, 17]
Mouse	BMMSC	I.V.	2x10^6	[18]
Mouse	BMMSC	I.V.	1-2x10^7	[19]
Mouse	BMMSC	I.V.	3x10^7	[20]
Mouse	CPMSC	I.V.	2x10^6	[21]
Mouse	EnSC	I.V.	1x10^6	[22, 23]
Mouse	EnSC	I.V.	2x10^6	[24, 25]
Mouse	EnSC	Local	1x10^4	[26]
Mouse	ESC	I.V.	5x10^6	[27]
Mouse	ESC	I.V.	1x10^6	[16]
Mouse	FGSC	I.V.	1x10^4	[1]
Mouse	LMSC	I.V.	1x10^6	[28], NCT03877471
Mouse	OGSC	I.V.	1x10^7	[29]
Mouse	SMSC	I.V.	2x10^6	[30]
Mouse	PDMSC	I.V.	1x10^6	[31, 32, 33, 34]
Mouse	UCMSC	I.V.	2x10^5	[35], NCT02644447
Mouse	UCMSC	I.V.	5x10^5	[36]
Mouse	UCMSC	I.V.	1x10^6	[12, 37, 38, 39, 40]
Rat	ADSC	I.V.	4x10^6	[41, 42]
Rat	ADSC	I.V.	5x10^6	[43]

		Local	$1x10^6$	[44]
			$2x10^6$	[45, 46], NCT01853501
	AEC	I.V. and Local	$2x10^6$,$4x10^{6,}$	[47]
	AMSC	I.V.	$5x10^6$	[43]
		I.V. and Local	$4x10^6$	[48]
		Local	$6-10x10^6$	[49]
	BMMSC	I.P.	$2x10^6$	[50]
			$4x10^6$	[51]
		I.V.	$1x10^6$	[52]
			$4x10^6$	[53, 54]
		Local	$1x10^6$	[55]
			$2x10^6$	[56]
	MenSC	I.V.	$1x10^6$	[57]
	PD-MSC	I.V.	$5x10^5$	[58, 59, 60]
		Local	$1x10^5$, $1x10^5$	[61]
			$1x10^5$, $1x10^6$	[62]
	UCMSC	I.V.	$1x10^6$	[63, 64]
			$2x10^6$	[65]
			$2.5x10^5$, $1x10^6$, $4x10^6$	[66]
			$5x10^6$ (2times)	[67]
		Local	$5x10^5$	[68]
			$2x10^6$	[69]

I.P. Intraperitoneal injection*, I.V.* Intravenous injection*, Local* intra ovarian injection*, BMMSC* bone marrow mesenchymal stem cells, *ADSC*Adipose-derived mesenchymal stem cells, *CPMSC* chorionic plate derived mesenchymal stem cells, *UCMSC* umbilical cord mesenchymal stem cells, *AEC* amnion epithelial cells, *AMSC* human amniotic mesenchymal stem cells, *AFSC* Amniotic fluid mesenchymal stem cells, *EnSC,* Endometrial stem cells, ESC embryonic stem cells, *LMSC* Human fetal liver MSCs, *SMSC*mSkin derived MSCs, *OGSC*hiPSC-derived OGLCS Ovarian granulosa like cells, *FGSC* Female Germline Stem cells, *PD-MSC* Placenta derived mesenchymal stem cell, *MenSCs* menstrual blood stem cells

Supplementary references

1. Terraciano P, Garcez T, Ayres L, Durli I, Baggio M et al.: Cell therapy for chemically induced ovarian failure in mice. Stem Cells Int2014; 2014:720753.
2. Sun M, Wang S, Li Y, Yu L, Gu F et al.: Adipose-derived stem cells improved mouse ovary function after chemotherapy-induced ovary failure. Stem Cell Res Ther2013; 4(4):80.
3. Yao X, Guo Y, Wang Q, Xu M, Zhang Q et al.: The Paracrine Effect of Transplanted Human Amniotic Epithelial Cells on Ovarian Function Improvement in a Mouse Model of Chemotherapy-Induced Primary Ovarian Insufficiency. Stem Cells Int2016; 2016:4148923.
4. Wang F, Wang L, Yao X, Lai D, Guo L: Human amniotic epithelial cells can differentiate into granulosa cells and restore folliculogenesis in a mouse model of chemotherapy-induced premature ovarian failure. Stem Cell Res Ther2013; 4(5):124.
5. Zhang Q, Huang Y, Sun J, Gu T, Shao X et al.: Immunomodulatory effect of human amniotic epithelial cells on restoration of ovarian function in mice with autoimmune ovarian disease. Acta Biochim Biophys Sin (Shanghai)2019; 51(8):845-855.
6. Liu T, Qin W, Huang Y, Zhao Y, Wang J: Induction of estrogen-sensitive epithelial cells derived from human-induced pluripotent stem cells to repair ovarian function in a chemotherapy-induced mouse model of premature ovarian failure. DNA Cell Biol2013; 32(12):685-698.
7. Zhang Q, Bu S, Sun J, Xu M, Yao X et al.: Paracrine effects of human amniotic epithelial cells protect against chemotherapy-induced ovarian damage. Stem Cell Res Ther2017; 8(1):270.
8. Liu T, Huang Y, Guo L, Cheng W, Zou G: CD44+/CD105+ human amniotic fluid mesenchymal stem cells survive and proliferate in the ovary long-term in a mouse model of chemotherapy-induced premature ovarian failure. Int J Med Sci2012; 9(7):592-602.
9. Lai D, Wang F, Chen Y, Wang L, Wang Y et al.: Human amniotic fluid stem cells have a potential to recover ovarian function in mice with chemotherapy-induced sterility. BMC Dev Biol2013; 13:34.
10. Xiao GY, Liu IH, Cheng CC, Chang CC, Lee YH et al.: Amniotic fluid stem cells prevent follicle atresia and rescue fertility of mice with premature ovarian failure induced by chemotherapy. PLoS One2014; 9(9):e106538.
41. Liu R, Zhang X, Fan Z, Wang Y, Yao G et al.: Human amniotic mesenchymal stem cells improve the follicular microenvironment to recover ovarian function in premature ovarian failure mice. Stem Cell Res Ther2019; 10(1):299.
12. Pan Y, Zhang L, Zhang X, Hu C, Liu R: Biological and biomechanical analysis of two types of mesenchymal stem cells for intervention in chemotherapy-induced ovarian dysfunction. Arch Gynecol Obstet2017; 295(1):247-252.
13. Mohamed SA, Shalaby SM, Abdelaziz M, Brakta S, Hill WD et al.: Human Mesenchymal Stem Cells Partially Reverse Infertility in Chemotherapy-Induced Ovarian Failure. Reprod Sci2018; 25(1):51-63.
14. Liu T, Wang S, Li Q, Huang Y, Chen C et al.: Telocytes as potential targets in a cyclophosphamide-induced animal model of premature ovarian failure. Mol Med Rep2016; 14(3):2415-2422.
15. Badawy A, Sobh MA, Ahdy M, Abdelhafez MS: Bone marrow mesenchymal stem cell repair of cyclophosphamide-induced ovarian insufficiency in a mouse model. Int J Womens Health2017; 9:441-447.
16. Bahrehbar K, Rezazadeh Valojerdi M, Esfandiari F, Fathi R, Hassani SN et al.: Human embryonic stem cell-derived mesenchymal stem cells improved premature ovarian failure. World J Stem Cells2020; 12(8):857-878.
17. Herraiz S, Buigues A, Diaz-Garcia C, Romeu M, Martinez S et al.: Fertility rescue and ovarian follicle growth promotion by bone marrow stem cell infusion. Fertil Steril2018; 109(5):908-918 e902.
18. Peng J, Xiao N, Cheng L: [Therapeutic potential of BMSCs for premature ovarian failure in mice]. Zhong Nan Da Xue Xue Bao Yi Xue Ban2018; 43(1):7-13.
19. Ghadami M, El-Demerdash E, Zhang D, Salama SA, Binhazim AA et al.: Bone marrow transplantation restores follicular maturation and steroid hormones production in a mouse model for primary ovarian failure. PLoS One2012; 7(3):e32462.
20. Lee HJ, Selesniemi K, Niikura Y, Niikura T, Klein R et al.: Bone marrow transplantation generates immature oocytes and rescues long-term fertility in a preclinical mouse model of chemotherapy-induced premature ovarian failure. J Clin Oncol2007; 25(22):3198-3204.
21. Li J, Yu Q, Huang H, Deng W, Cao X et al.: Human chorionic plate-derived mesenchymal stem cells transplantation restores ovarian function in a chemotherapy-induced mouse model of premature ovarian failure. Stem Cell Res Ther2018; 9(1):81.
22. Feng P, Li P, Tan J: Human Menstrual Blood-Derived Stromal Cells Promote Recovery of Premature Ovarian Insufficiency Via Regulating the ECM-Dependent FAK/AKT Signaling. Stem Cell Rev

Rep2019; 15(2):241-255.
23. Guo F, Xia T, Zhang Y, Ma X, Yan Z et al.: Menstrual blood derived mesenchymal stem cells combined with Bushen Tiaochong recipe improved chemotherapy-induced premature ovarian failure in mice by inhibiting GADD45b expression in the cell cycle pathway. Reprod Biol Endocrinol2019; 17(1):56.
24. Lai D, Wang F, Yao X, Zhang Q, Wu X et al.: Human endometrial mesenchymal stem cells restore ovarian function through improving the renewal of germline stem cells in a mouse model of premature ovarian failure. J Transl Med2015; 13:155.
25. Wang Z, Wang Y, Yang T, Li J, Yang X: Study of the reparative effects of menstrual-derived stem cells on premature ovarian failure in mice. Stem Cell Res Ther2017; 8(1):11.
26. Liu T, Huang Y, Zhang J, Qin W, Chi H et al.: Transplantation of human menstrual blood stem cells to treat premature ovarian failure in mouse model. Stem Cells Dev2014; 23(13):1548-1557.
27. Yoon SY, Yoon JA, Park M, Shin EY, Jung S et al.: Recovery of ovarian function by human embryonic stem cell-derived mesenchymal stem cells in cisplatin-induced premature ovarian failure in mice. Stem Cell Res Ther2020; 11(1):255.
28. Huang B, Qian C, Ding C, Meng Q, Zou Q et al.: Fetal liver mesenchymal stem cells restore ovarian function in premature ovarian insufficiency by targeting MT1. Stem Cell Res Ther2019; 10(1):362.
29. Liu T, Li Q, Wang S, Chen C, Zheng J: Transplantation of ovarian granulosa-like cells derived from human induced pluripotent stem cells for the treatment of murine premature ovarian failure. Mol Med Rep2016; 13(6):5053-5058.
30. Lai D, Wang F, Dong Z, Zhang Q: Skin-derived mesenchymal stem cells help restore function to ovaries in a premature ovarian failure mouse model. PLoS One2014; 9(5):e98749.
31. Li H, Zhao W, Wang L, Luo Q, Yin N et al.: Human placenta-derived mesenchymal stem cells inhibit apoptosis of granulosa cells induced by IRE1alpha pathway in autoimmune POF mice. Cell Biol Int2019; 43(8):899-909.
32. Yin N, Zhao W, Luo Q, Yuan W, Luan X et al.: Restoring Ovarian Function With Human Placenta-Derived Mesenchymal Stem Cells in Autoimmune-Induced Premature Ovarian Failure Mice Mediated by Treg Cells and Associated Cytokines. Reprod Sci2018; 25(7):1073-1082.
33. Zhang H, Luo Q, Lu X, Yin N, Zhou D et al.: Effects of hPMSCs on granulosa cell apoptosis and AMH expression and their role in the restoration of ovary function in premature ovarian failure mice. Stem Cell Res Ther2018; 9(1):20.
34. Yin N, Wang Y, Lu X, Liu R, Zhang L et al.: hPMSC transplantation restoring ovarian function in premature ovarian failure mice is associated with change of Th17/Tc17 and Th17/Treg cell ratios through the PI3K/Akt signal pathway. Stem Cell Res Ther2018; 9(1):37.
35. Yang Y, Lei L, Wang S, Sheng X, Yan G et al.: Transplantation of umbilical cord-derived mesenchymal stem cells on a collagen scaffold improves ovarian function in a premature ovarian failure model of mice. In Vitro Cell Dev Biol Anim2019; 55(4):302-311.
36. Mohamed SA, Shalaby S, Brakta S, Elam L, Elsharoud A et al.: Umbilical Cord Blood Mesenchymal Stem Cells as an Infertility Treatment for Chemotherapy Induced Premature Ovarian Insufficiency. Biomedicines2019; 7(1).
37. Shen J, Cao D, Sun JL: Ability of human umbilical cord mesenchymal stem cells to repair chemotherapy-induced premature ovarian failure. World J Stem Cells2020; 12(4):277-287.
38. Jalalie L, Rezaee MA, Rezaie MJ, Jalili A, Raoofi A et al.: Human umbilical cord mesenchymal stem cells improve morphometric and histopathologic changes of cyclophosphamide-injured ovarian follicles in mouse model of premature ovarian failure. Acta Histochem2021; 123(1):151658.
39. Jalalie L, Rezaie MJ, Jalili A, Rezaee MA, Vahabzadeh Z et al.: Distribution of the CM-Dil-Labeled Human Umbilical Cord Vein Mesenchymal Stem Cells Migrated to the Cyclophosphamide-Injured Ovaries in C57BL/6 Mice. Iran Biomed J2019; 23(3):200-208.
40. Wang S, Yu L, Sun M, Mu S, Wang C et al.: The therapeutic potential of umbilical cord mesenchymal stem cells in mice premature ovarian failure. Biomed Res Int2013; 2013:690491.
41. Ling L, Feng X, Wei T, Wang Y, Wang Y et al.: Effects of low-intensity pulsed ultrasound (LIPUS)-pretreated human amnion-derived mesenchymal stem cell (hAD-MSC) transplantation on primary ovarian insufficiency in rats. Stem Cell Res Ther2017; 8(1):283.
42. Ling L, Feng X, Wei T, Wang Y, Wang Y et al.: Human amnion-derived mesenchymal stem cell (hAD-MSC) transplantation improves ovarian function in rats with premature ovarian insufficiency (POI) at least partly through a paracrine mechanism. Stem Cell Res Ther2019; 10(1):46.
43. Fouad H, Sabry D, Elsetohy K, Fathy N: Therapeutic efficacy of amniotic membrane stem cells and adipose tissue stem cells in rats with chemically induced ovarian failure. J Adv Res2016; 7(2):233-241.
44. Vural B, Duruksu G, Vural F, Gorguc M, Karaoz E: Effects of VEGF (+) Mesenchymal Stem Cells

and Platelet-Rich Plasma on Inbred Rat Ovarian Functions in Cyclophosphamide-Induced Premature Ovarian Insufficiency Model. Stem Cell Rev Rep2019; 15(4):558-573.
45. Su J, Ding L, Cheng J, Yang J, Li X et al.: Transplantation of adipose-derived stem cells combined with collagen scaffolds restores ovarian function in a rat model of premature ovarian insufficiency. Hum Reprod2016; 31(5):1075-1086.
46. Takehara Y, Yabuuchi A, Ezoe K, Kuroda T, Yamadera R et al.: The restorative effects of adipose-derived mesenchymal stem cells on damaged ovarian function. Lab Invest2013; 93(2):181-193.
47. Zhang Y, Ouyang X, You S, Zou H, Shao X et al.: Effect of human amniotic epithelial cells on ovarian function, fertility and ovarian reserve in primary ovarian insufficiency rats and analysis of underlying mechanisms by mRNA sequencing. Am J Transl Res2020; 12(7):3234-3254.
48. Feng X, Ling L, Zhang W, Liu X, Wang Y et al.: Effects of Human Amnion-Derived Mesenchymal Stem Cell (hAD-MSC) Transplantation In Situ on Primary Ovarian Insufficiency in SD Rats. Reprod Sci2020; 27(7):1502-1512.
49. Gan L, Duan H, Xu Q, Tang YQ, Li JJ et al.: Human amniotic mesenchymal stromal cell transplantation improves endometrial regeneration in rodent models of intrauterine adhesions. Cytotherapy2017; 19(5):603-616.
50. Besikcioglu HE, Saribas GS, Ozogul C, Tiryaki M, Kilic S et al.: Determination of the effects of bone marrow derived mesenchymal stem cells and ovarian stromal stem cells on follicular maturation in cyclophosphamide induced ovarian failure in rats. Taiwan J Obstet Gynecol2019; 58(1):53-59.
51. Kilic S, Pinarli F, Ozogul C, Tasdemir N, Naz Sarac G et al.: Protection from cyclophosphamide-induced ovarian damage with bone marrow-derived mesenchymal stem cells during puberty. Gynecol Endocrinol2014; 30(2):135-140.
52. Gabr H, Rateb MA, El Sissy MH, Ahmed Seddiek H, Ali Abdelhameed Gouda S: The effect of bone marrow-derived mesenchymal stem cells on chemotherapy induced ovarian failure in albino rats. Microsc Res Tech2016; 79(10):938-947.
53. Liu J, Zhang H, Zhang Y, Li N, Wen Y et al.: Homing and restorative effects of bone marrow-derived mesenchymal stem cells on cisplatin injured ovaries in rats. Mol Cells2014; 37(12):865-872.
54. Zhang Q, Xu M, Yao X, Li T, Wang Q et al.: Human amniotic epithelial cells inhibit granulosa cell apoptosis induced by chemotherapy and restore the fertility. Stem Cell Res Ther2015; 6(1):152.
55. Fu X, He Y, Wang X, Peng D, Chen X et al.: Overexpression of miR-21 in stem cells improves ovarian structure and function in rats with chemotherapy-induced ovarian damage by targeting PDCD4 and PTEN to inhibit granulosa cell apoptosis. Stem Cell Res Ther2017; 8(1):187.
56. Fu X, He Y, Xie C, Liu W: Bone marrow mesenchymal stem cell transplantation improves ovarian function and structure in rats with chemotherapy-induced ovarian damage. Cytotherapy2008; 10(4):353-363.
57. Noory P, Navid S, Zanganeh BM, Talebi A, Borhani-Haghighi M et al.: Human Menstrual Blood Stem Cell-Derived Granulosa Cells Participate in Ovarian Follicle Formation in a Rat Model of Premature Ovarian Failure In Vivo. Cell Reprogram2019; 21(5):249-259.
58. Choi JH, Seok J, Lim SM, Kim TH, Kim GJ: Microenvironmental changes induced by placenta-derived mesenchymal stem cells restore ovarian function in ovariectomized rats via activation of the PI3K-FOXO3 pathway. Stem Cell Res Ther2020; 11(1):486.
59. Cho J, Kim TH, Seok J, Jun JH, Park H et al.: Vascular remodeling by placenta-derived mesenchymal stem cells restores ovarian function in ovariectomized rat model via the VEGF pathway. Lab Invest2021; 101(3):304-317.
60. Seok J, Park H, Choi JH, Lim JY, Kim KG et al.: Placenta-Derived Mesenchymal Stem Cells Restore the Ovary Function in an Ovariectomized Rat Model via an Antioxidant Effect. Antioxidants (Basel)2020; 9(7).
61. Park H, Seok J, You JH, Lee DH, Lim JY et al.: Can a Large Number of Transplanted Mesenchymal Stem Cells Have an Optimal Therapeutic Effect on Improving Ovarian Function? Int J Mol Sci2022; 23(24).
62. Kim TH, Choi JH, Jun Y, Lim SM, Park S et al.: 3D-cultured human placenta-derived mesenchymal stem cell spheroids enhance ovary function by inducing folliculogenesis. Sci Rep2018; 8(1):15313.
63. Song D, Zhong Y, Qian C, Zou Q, Ou J et al.: Human Umbilical Cord Mesenchymal Stem Cells Therapy in Cyclophosphamide-Induced Premature Ovarian Failure Rat Model. Biomed Res Int2016; 2016:2517514.
64. Li J, Mao Q, He J, She H, Zhang Z et al.: Human umbilical cord mesenchymal stem cells improve the reserve function of perimenopausal ovary via a paracrine mechanism. Stem Cell Res Ther2017; 8(1):55.
65. Lu X, Bao H, Cui L, Zhu W, Zhang L et al.: hUMSC transplantation restores ovarian function in POI rats by inhibiting autophagy of theca-interstitial cells via the AMPK/mTOR signaling pathway. Stem Cell

Res Ther2020; 11(1):268.
66. Wang Z, Wei Q, Wang H, Han L, Dai H et al.: Mesenchymal Stem Cell Therapy Using Human Umbilical Cord in a Rat Model of Autoimmune-Induced Premature Ovarian Failure. Stem Cells Int2020; 2020:3249495.
67. Zheng Q, Fu X, Jiang J, Zhang N, Zou L et al.: Umbilical Cord Mesenchymal Stem Cell Transplantation Prevents Chemotherapy-Induced Ovarian Failure via the NGF/TrkA Pathway in Rats. Biomed Res Int2019; 2019:6539294.
68. Zhang X, Zhang L, Li Y, Yin Z, Feng Y et al.: Human umbilical cord mesenchymal stem cells (hUCMSCs) promotes the recovery of ovarian function in a rat model of premature ovarian failure (POF). Gynecol Endocrinol2021; 37(4):353-357.
69. Elfayomy AK, Almasry SM, El-Tarhouny SA, Eldomiaty MA: Human umbilical cord blood-mesenchymal stem cells transplantation renovates the ovarian surface epithelium in a rat model of premature ovarian failure: Possible direct and indirect effects. Tissue Cell2016; 48(4):370-382.
